# Supplementary material for: The Relationship between Obesity-Related Factors and Graves’ Orbitopathy: A Pilot Study
Source: Medicina (Kaunas). 2022 Nov 29;58(12):1748. doi: 10.3390/medicina58121748 (PMC9784517; doi:10.3390/medicina58121748)
Supplement: Supplementary file 1 [file medicina-58-01748-s001.zip › Supplement Table S1 .pdf]

Supplement Table S1. Associations between risk factors and Graves' orbitopathy among patients with Graves' disease (N = 84) in multivariate logistic regression analysis

	Odds ratio	95% confidence interval for odds ratio		*P*
		Lower	Upper	
Age	1.019	0.975	1.065	0.398
Gender(male)	3.453	0.842	14.162	0.085
Smoking	3.762	0.901	15.710	0.069
Titer of TSHR antibodies	1.020	1.000	1.039	0.047
Steroid usage	1570930407.575	0.000	.	0.999
HOMA-IR	1.985	1.046	3.764	0.036

TSHR, thyroid-stimulating hormone receptor; HOMA-IR, homeostasis model assessment-estimated insulin resistance

Dataset: 20220710 excel
